# chemalot and chemalot_knime: Command line programs as workflow tools for drug discovery

**DOI:** 10.1186/s13321-017-0228-9

**Published:** 2017-06-12

**Authors:** Man-Ling Lee, Ignacio Aliagas, Jianwen A. Feng, Thomas Gabriel, T. J. O’Donnell, Benjamin D. Sellers, Bernd Wiswedel, Alberto Gobbi

**Affiliations:** 1 Discovery Chemistry, Genentech Inc., 1 DNA Way, South San Francisco, CA 94080 USA; 2 Denali Therapeutics, 151 Oyster Point Blvd, South San Francisco, CA 94080 USA; 3 KNIME.com AG, Technoparkstrasse 1, 8005 Zurich, Switzerland; 4 gNova Scientific Software, San Diego, CA 92103 USA

**Keywords:** Command line program, Substructure identification, Property calculation, SAR, QSPR model, Conformation analysis, Strain energy analysis, Dynamic KNIME node generation

## Abstract

**Background:**

Analyzing files containing chemical information is at the core of cheminformatics. Each analysis may require a unique workflow. This paper describes the chemalot and chemalot_knime open source packages. Chemalot is a set of command line programs with a wide range of functionalities for cheminformatics. The chemalot_knime package allows command line programs that read and write SD files from stdin and to stdout to be wrapped into KNIME nodes. The combination of chemalot and chemalot_knime not only facilitates the compilation and maintenance of sequences of command line programs but also allows KNIME workflows to take advantage of the compute power of a LINUX cluster.

**Results:**

Use of the command line programs is demonstrated in three different workflow examples: (1) A workflow to create a data file with project-relevant data for structure–activity or property analysis and other type of investigations, (2) The creation of a quantitative structure–property-relationship model using the command line programs via KNIME nodes, and (3) The analysis of strain energy in small molecule ligand conformations from the Protein Data Bank database.

**Conclusions:**

The chemalot and chemalot_knime packages provide lightweight and powerful tools for many tasks in cheminformatics. They are easily integrated with other open source and commercial command line tools and can be combined to build new and even more powerful tools. The chemalot_knime package facilitates the generation and maintenance of user-defined command line workflows, taking advantage of the graphical design capabilities in KNIME.

**Graphical abstract:**
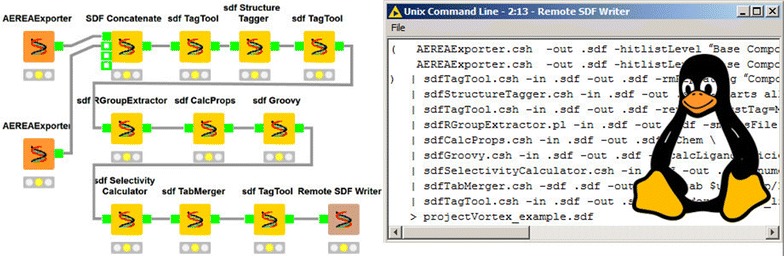
Example KNIME workflow with chemalot nodes and the corresponding command line pipe

**Electronic supplementary material:**

The online version of this article (doi:10.1186/s13321-017-0228-9) contains supplementary material, which is available to authorized users.

## Background

In the fields of computational chemistry and cheminformatics, data management and analysis require the sequential application of multiple methods and algorithms. For example, in a hierarchical docking workflow, two-dimensional molecular structures may first be converted into a set of three-dimensional models using a conformer generation tool. The conformers may then be pre-screened with a fast pharmacophore matching tool to remove conformers that will not be able to make the required interactions. The reduced set of conformers may subsequently be docked to a protein structure and scored with more resource-intensive, but more accurate methods. To keep up with project teams’ demand, a flexible development environment that allows the quick assembly of cheminformatics and structure-based design modules has become indispensable for prototyping, developing, and deploying computational workflows. Example workflow platforms include UNIX shell scripting with pipes [1], open source visual workflow tools such as KNIME [2] and ORANGE [3], as well as commercial applications such as Pipeline Pilot [4]. The power of each tool depends on the strength and robustness of the available modules and the flexibility and ease with which the modules can be combined and extended.

**Fig. 1:**
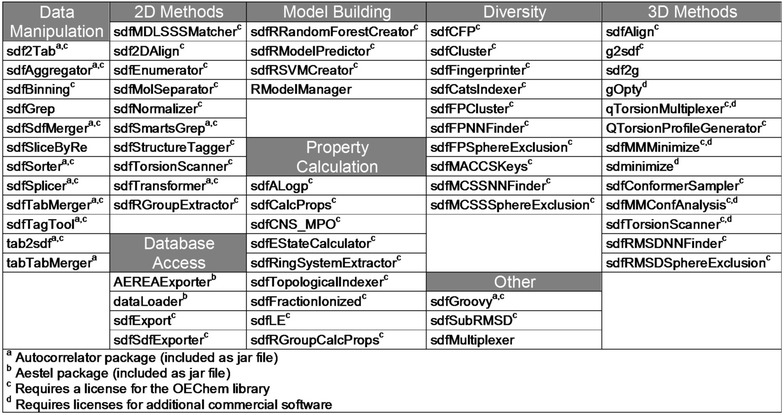
List of command line programs available in the chemalot package. For a short description of each command line tool the reader is referred to the GitHub website

This paper introduces chemalot, a set of command line programs for cheminformatics and structure-based design developed at Genentech that can be used for a wide variety of tasks. These command line programs extend a set of previously released command line programs from the Autocorrelator package [5] and include published programs for database access and diversity analysis [6, 7]. The full list of command line programs is given in Fig. 1. They provide diverse functionalities such as data manipulation and filtering, database access, property calculation, model building and 3D structure analysis. The programs take advantage of integration with other software such as R [8], Gaussian [9], and OpenEye toolkits [10]. The tasks performed by the command line programs can be as simple as renaming data fields in the input file or as complex as performing a 3D conformational analysis. Most of chemalot command line programs read and write Structure Data (SD) files [11]. These programs can be combined with each other and with command line programs from open source or commercial cheminformatics packages [10–14] to create complex UNIX pipelines. In addition, a small number of the chemalot command line programs read and write tab-delimited files to better handle spreadsheet data.

Implementing workflows using UNIX scripts and pipes of command line programs is very lightweight while still powerful. The UNIX infrastructure supports scheduled execution with cron [15] as well as distributed execution on high performance computing clusters with tools like LSF [16] and PBS [17]. The sdfMultiplexer program allows for the parallel execution of workflows over multiple cores or compute nodes by seamlessly splitting the input stream and combining the output streams. Parallelization with sdfMultiplexer is only possible for workflows in which a computation is independently applied to each record. These features make the UNIX platform and command line programs ideal building blocks for simple and complex cheminformatics workflows.

On the other hand, UNIX scripts and pipes are hard to debug compared to workflows developed in graphical workflow tools such as KNIME, ORANGE, and Pipeline Pilot. For this reason, KNIME and Pipeline Pilot have become very popular as they provide a powerful set of modules as well as a graphical user interface for developing workflows. A useful feature of these visual programming environments is that workflows can be executed one module at a time. This enables inspection of the output of each module and thus debugging is greatly simplified. However, there are some downsides to using visual workflow environments. The execution of a workflow may incur a significant start-up cost because the execution environment needs to be initialized. When executing on multiple nodes in a cluster the initialization needs to happen on each node. While the environment can be pre-started on each node, when no workflows are executing, the resources may be reserved and unavailable for other computations.

To leverage the benefits of the lightweight execution of command line programs in UNIX pipes and the debugging power of graphical workflow software, we have implemented a framework within the open source KNIME Analytics Platform that exposes command line programs as KNIME nodes. By using the DynamicNodeFactory class in the KNIME API, the framework can generate KNIME nodes from a simple XML configuration. Thus, given a command line program that reads and writes SD files, the creation of a new chemalot_knime node in KNIME takes only 5 lines in the XML file. Since the standard KNIME nodes only handle data in KNIME table format, two data format conversion nodes were implemented. They allow the integration of command line tools with other nodes available in KNIME, thus providing access to high performance computing clusters from within KNIME workflows. This also allows the integration with cheminformatics nodes from other open source packages such as the RDKit [18], CDK [19] and Vernalis [20] KNIME nodes. The chemalot_knime nodes not only output the processed data; they also compile an executable UNIX command corresponding to the given node sequence. Since the chemalot_knime nodes use UNIX pipes and do not rely on the workflow environment, the command can be copied and pasted into a UNIX shell script and executed in any UNIX computing environment, thus avoiding the startup costs mentioned previously.

We first provide a conceptional overview of the implementation of the chemalot command line programs by describing three representative programs and the KNIME framework for the dynamic node generation. Then, we will showcase three example workflows and their use at Genentech: (1) the creation of “project reports” visualized with Vortex [21], (2) the creation of statistical prediction models using the Random Forest machine learning method [22, 23], and (3) the implementation of a method to estimate the strain of 3D conformations of small molecules.

The command line programs are available in the chemalot package on GitHub [24] under the Apache Open Source license. The chemalot_knime node framework is available in the chemalot_knime package on GitHub [25] under the GNU General Public License version 3. Most of the command line tools require a license for the commercial OEChem library and additional commercial software is required for specific commands.

## Implementation

The goal of this section is to give the reader an understanding of the program design concept. Besides descriptions of the implementation details, this section also describes the context in which the command line programs presented in this section can be used.

### Command line programs

Most of the command line programs read and write molecular structures and associated data, e.g. names, properties, and activity data, in SD format. This format was chosen because its widespread use enables the integration with other open source and commercial command line programs. A few chemalot programs read and write other formats such as tab-separated and Gaussian formats to enable integration with other software packages.

The command line programs can be divided into two groups, Java programs and scripts. Molecule structure related and database dependent algorithms are mostly implemented as Java programs. The Java version of the commercial OpenEye OEChem toolkit [10] is used to perform cheminformatics algorithms such as substructure matching and to navigate the molecular structural graph as well as to parse SD files. Scripts frequently combine other command line programs in order to perform a common sequence of computations or to implement novel methods. A detailed description of how to use each program can be accessed by executing each program with the “-h” option. To showcase the distinction of Java programs and scripts, we will discuss three command line programs in more detail below: sdfStructureTagger, sdfMMConfAnalysis, and sdfCalcProps.

#### sdfStructureTagger: a Java program example

Drug discovery teams classify compounds into chemical series defined by a common core or scaffold. In reporting applications, the given series names are displayed along with the chemical structure to facilitate communication with non-chemists or to analyze structure activity relationship (SAR) trends within a series. Scaffolds can be specific substructures or more generic definitions, e.g. multiple scaffolds with similar attachment vectors.

The program sdfStructureTagger is used to “tag” compounds based on chemical scaffolds which the user defines using the SMARTS language [26]. By “tagging”, we mean to add a data field to the SD output stream that classifies a compound as a member of a given series. Examples could be “Azaindole” or “PhenylAmide Series”. A tab-delimited file with SMARTS, tag name, and tag set name is required as input. The tag set name allows a user to create sets of SMARTS, e.g. groups of main and sub series scaffold definitions in a single file. Each sdfStructureTagger execution can only use one set and the SMARTS are applied to an input molecule in the given order. If a molecule matches a SMARTS scaffold definition, the associated tag name is added to the molecule’s metadata in the SD file. Depending on the specification, the tag of the first matching SMARTS, all matching SMARTS, or both are added to the SD file. The order dependent application of the SMARTS and the output of the first match enable the user to assign input molecules into desired compound series without having to compile complex SMARTS. Having multiple SMARTS with the same tag name or placing the SMARTS in appropriate order are effective alternatives.

The sdfStructureTagger command line program is implemented in Java and uses the OEChem API for SD file parsing and substructure matching. The Apache Commons CLI library [27] is used to parse the command line options. The Java program is wrapped in a small C-shell script that sets the correct environment settings before the program execution. The Java code is separated into two classes, SDFStructureTagger.java and StructureTagger.java. SDFStructureTagger.java contains code to parse the command line parameter values as well as the content of the SMARTS and SD file. Its task is to pass each input molecule object to a StructureTagger instance. StructureTagger.java encapsulates the structure matching code. Most of the Java-based command line programs in the package [28] adopt a similar separation of the command line parsing and the algorithmic code. This facilitates the reuse of the code for other applications e.g. web services.

#### sdfMMConfAnalysis: a workflow script example

**Fig. 2:**
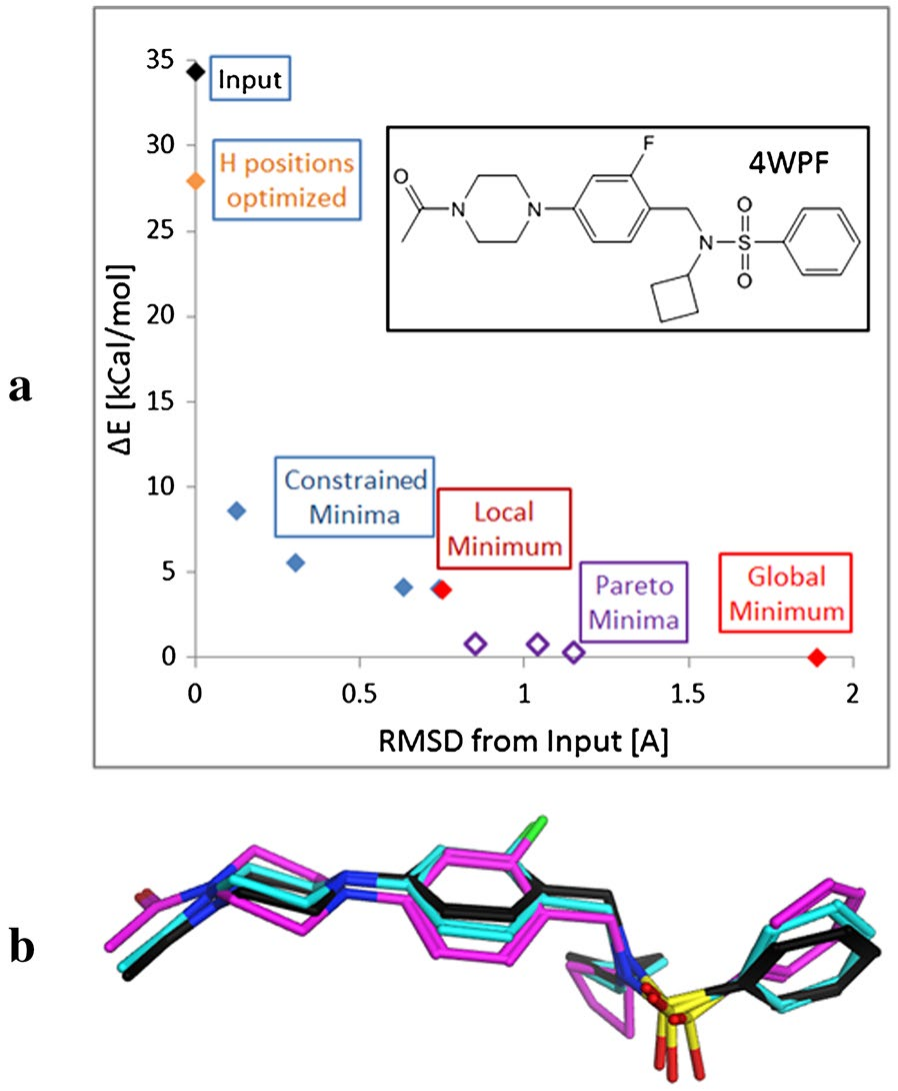
Conformational analysis of the sulfonamide ligand in the 4WPF PDB crystal structure performed with sdfMMConfAnalysis. **a** Profile of conformations returned. RMSD values on the x axis are relative to the input conformation. Energies on the y axis are relative to the global minimum. **b** Three conformations from the conformation analysis. The input conformation is in *black*, the constraint minimum at 0.3 Å from the input is in *blue* and the Pareto minimum at 0.8 Å from the input is shown in *pink*

The internal potential (strain) energy of a drug molecule is a key component of the binding affinity for a drug to a protein. It constitutes the unfavorable increase in energy of small molecule in solution when adopting the conformation in the binding site. No interactions with the protein are taken into account. The sdfMMConfAnalysis program follows a procedure similar to that first described by Boström et al. [29]. Given a crystallographic binding conformation or a three dimensional computational binding hypothesis, the program identifies the global minimum conformation, the local minimum closest to the input conformation as well as four intermediate conformations between the input and the local minimum. The relative energies of these conformations and their distance from the input conformation allow an estimation of the strain of the input conformation. All energies are computed with the MMFF94S [30] force field as implemented in SZYBKI [31] using the Sheffield solvation model [32]. The four conformations intermediate between the input and the local minimum are obtained by minimizing the input employing an additional flat bottom potential with flat bottom radius of 0.2, 0.6, 1.0 or 1.4 Å to constrain their deviation from the input conformation. These intermediate constrained minima together with the local minimum provide information about the height and slope of the potential energy surface around the input conformation (Fig. 2). A global minimum search is performed by enumerating the accessible conformations with OMEGA, sampling additional OH and NH rotors with sdfTorsionScanner, minimizing them with SZYBKI, and selecting the lowest energy conformation as the global minimum. If the minimized OMEGA conformational ensemble includes conformations that are Pareto optimal [33] with regards to low energy and small RMSD to the constrained minimized and global minimum conformations, they are also retained. The output file contains the input conformation, conformations from the four constrained minimizations, the local minimum, and the global minimum as well as any additional Pareto optimal local minima. All output structures are aligned to the input structure using sdfAlign that aligns structures by minimizing the RMSD. The energy profile and some of the conformations identified, starting from the crystallographic conformation of the sulfonamide ligand in the 4WPF structure [34], are shown in Fig. 2.

**Fig. 3:**
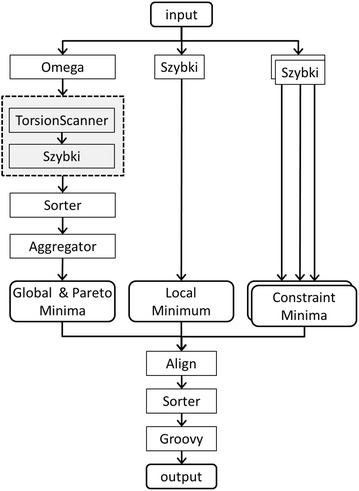
Conformational analysis workflow as implemented in sdfMMConfAnalysis. Depending on the command *line* options, the programs in the *dotted box* are executed in parallel using sdfMultiplexer

SdfMMConfAnalysis is implemented as a Perl script which wraps a set of command line tools and commercial applications. A diagram of the workflow is given in Fig. 3. Perl scripting is only used to parse the command line options and execute the sequence of piped programs, shown as rectangular boxes. To speed up the calculation, the minimization of the conformational ensemble generated by OMEGA can be parallelized. This is accomplished by executing the piped set of command line programs, highlighted by the dotted box in Fig. 3, using sdfMultiplexer. Even though up to 500 minimizations are performed for a full conformational analysis, on average the workflow takes about 20 s when executed on an 8 core computer.

#### sdfCalcProps: a workflow assembler program

The previous section shows that command line programs can be combined to create a new program that executes a predefined workflow. In this section, we describe the sdfCalcProps program that dynamically creates a workflow depending on the properties requested by the user. This task can be complicated as the calculation of one property might depend on other properties. For example, the familiar Rule of Five (RO5) model evaluates drug likeness of a molecule based on its number of hydrogen bond donors (NH + OH), acceptors (N + O), molecular weight (MW), and cLogP [35]. The dependencies result in a hierarchy of properties that requires calculators to be executed in a specific order. In this example, NH + OH, N + O, MW, and cLogP have to be computed before RO5 can be calculated. As the number of properties increases, tracking the dependencies hardcoded in a script would become unmanageable. For this reason, we devised a simple format to encode the dependencies in an XML file.

**Fig. 4:**
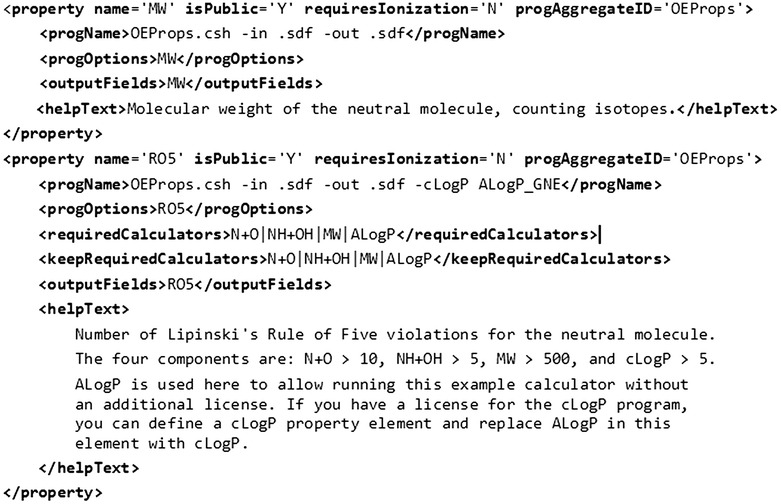
Property elements describing the MW and RO5 calculators in the sdfCalcProps XML configuration file

Figure 4 shows an XML snippet describing the MW and RO5 properties. Each property element describes how a program should be executed to create appropriate output(s). For a higher level property such as RO5, its dependencies are specified in the “requiredCalculators” field. Outputs from the “requiredCalculators” may be retained in the resulting output file by specifying “keepRequiredCalculators”. Many properties like MW, N + O and NH + OH are calculated using the same calculator (i.e. “OEProps”), but with different arguments. To ensure that the calculator is only called once, these properties are assigned the same “progAggregateID”.

**Fig. 5:**
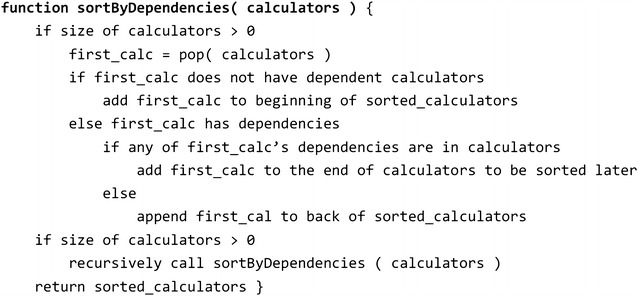
Pseudo code describing how dependencies are resolved by sdfCalcProps

When executing the sdfCalcProps program with a list of requested properties, sdfCalcProps determines the dependencies and creates a set of Calculator-Java objects for each property and its dependent properties. Calculator objects for properties having the same “progAggregateID” are merged, i.e. they are replaced with a single Calculator object. A recursive sorting places calculators with no dependencies at the beginning of the execution in order to avoid duplicate calculations. The pseudo code in Fig. 5 describes the recursive sorting algorithm. The sorted list of calculators is used to create a shell script that executes the calculators in the correct order.

Abstracting “property calculators” from the actual command line programs as well as separating the property calculator descriptions in an XML file from the compilation of command line text provides flexibility and facilitates the addition of new property calculators. The XML file describes the dependencies for all models used at Genentech, therefore sdfCalcProps can be considered as a “warehouse” of prediction models.

### chemalot_knime nodes

The chemalot_knime nodes allow the execution of any command line program that reads and writes SD files from within the KNIME Analytics Platform. The nodes are connected with each other via a custom KNIME port type, i.e. SDFCmdPortObject. Each chemalot_knime node can be executed on its own. In this case the associated command line program is started remotely via secure shell (ssh) and the contents of the input is sent as SD file to the standard input of the command line program. The node then captures the standard output of the program via ssh and makes it available on the output port of the node. The SDFCmdPortObject not only transfers data in SD file format, but also transfers the user configured command line options of all preceding nodes. This allows for a second execution mode with better performance in which a single “piped” UNIX command line is assembled from all nodes, but only executed at the end of the chemalot_knime node sequence. The corresponding command line text can be retrieved via the KNIME port view or via a KNIME flow variable and can be used in a shell script.

**Fig. 6:**
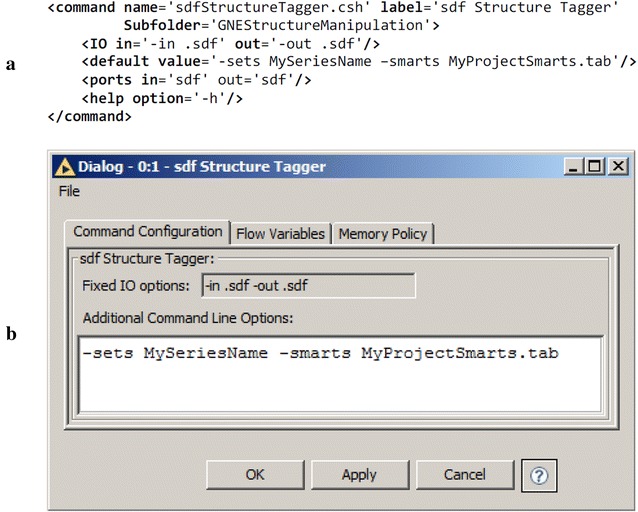
Example chemalot_knime node configuration. **a** XML snippet showing the definition of the sdfStructureTagger node and **b** the corresponding Command Configuration dialog generated by the dynamic node creation framework

Most chemalot_knime nodes are created using KNIME’s dynamic node creation framework [36]. The framework is used to generate chemalot_knime nodes from an XML file. Figure 6a shows the definition for the sdfStructureTagger node. The specification contains information (1) for organizing the node in the KNIME Node Repository (*label* and *Subfolder* attributes), (2) for help text generation (*help* element), (3) for indication of input and output port existence (*ports* element), and (4) for command text generation (*IO* and *default* elements). Figure 6b shows the corresponding configuration dialog. All program options except for the IO options are entered into the text box. Having a single text box greatly reduces the time needed to transfer program options from shell scripts. The “?” icon provides access to the help text of the given command line program.

**Fig. 7:**
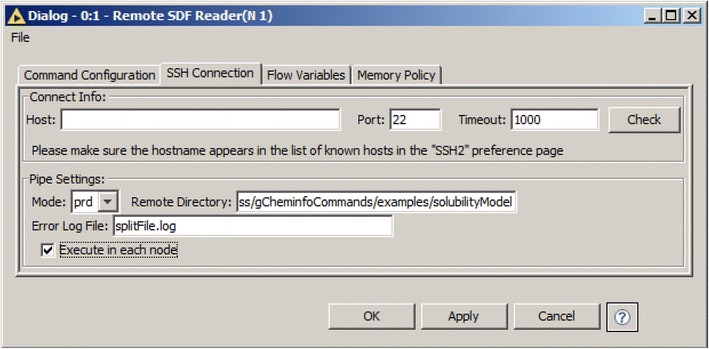
SSH settings tab in the configuration dialog of the chemalot_knime node

The sdfStructureTagger node has input and output SDFCmdPortObject ports and is therefore classified as a Processor node. The other two node types are Generator and Consumer nodes which have only output or only input SDFCmdPortObject ports, respectively. Generator nodes always constitute the start of a pipe and have an additional tab for specifying the ssh settings in their configuration dialog (Fig. 7). The “Remote Directory” input allows the user to specify an execution directory for all nodes in the sequence. File names specified as command line arguments are relative to the “Remote Directory”. The “Execute in each node” checkbox allows the user to defer the execution of the command line programs to the terminal Consumer node. If it is unchecked, the commands in the sequence are executed in parallel and, the data is transferred only at the beginning and end of the sequence. This provides the highest performance. If “Execute in each node” is checked, debugging the commands is simplified by enabling the inspection of the output and standard error of each node independently.

**Fig. 8:**
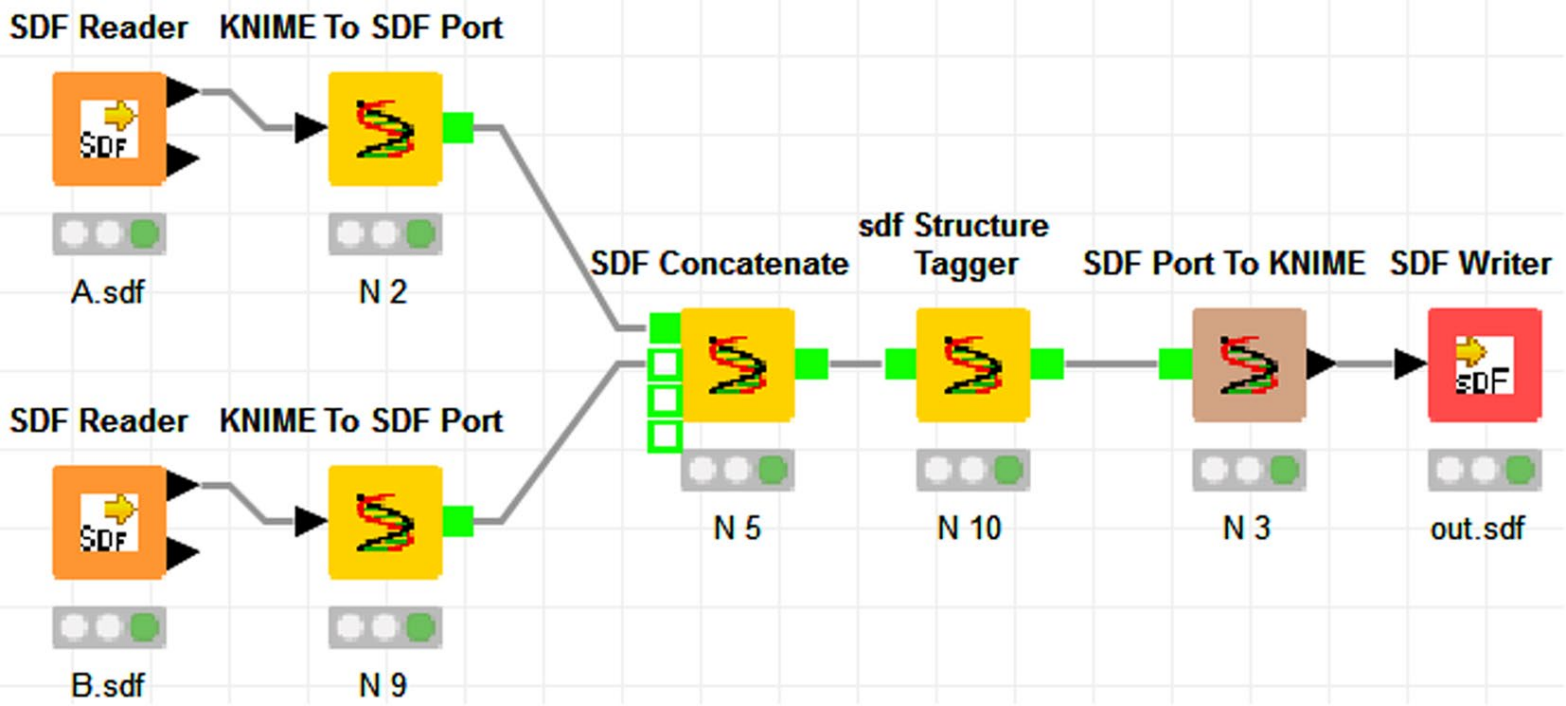
Example KNIME workflow showing the conversion and concatenation nodes

Additionally the chemalot_knime package provides two converter nodes that convert data in SD file format to the KNIME table format and vice versa. This enables integration between chemalot_knime nodes and standard KNIME nodes (cf. Fig. 8). We have also implemented the “SDF Concatenate” node to allow concatenation of multiple inputs.

## Results and discussion

The applications presented in this section demonstrate how the command line programs have been successfully used to support therapeutic project teams in various aspects of drug discovery at Genentech. The selected applications are essential tools for the day-to-day work of project team members. The “Results and discussion” section concludes with a summary of how command line programs enabled project teams at Denali Therapeutics to access data before the deployment of the research informatics infrastructure.

### Project Vortex sessions

Therapeutic project teams must be able to easily retrieve all relevant project data from a database and then be able to analyze and mine the data to make informed decisions. Instead of implementing a full-featured database application that includes both the data export and data mining user interface, we adopted a component-based approach: a set of chemalot command line programs serves as the backend for data export and processing while Vortex [21], a powerful, chemistry-aware commercial spreadsheet and plotting tool, serves as the frontend for data analysis. The interface between the backend and frontend is an SD file that can also be shared with research partners, whether they use Vortex or other visualization software (e.g. Spotfire [37] or DataWarrior [38]). Use of the SD file data format also provides the agility to integrate with other data analysis applications.

**Fig. 9:**
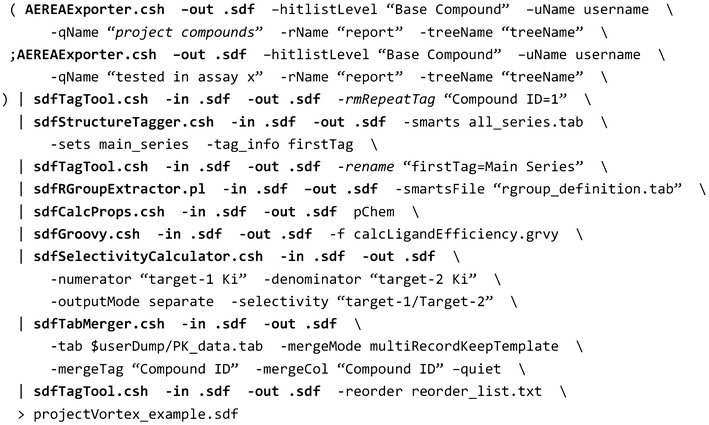
An example command line pipe for compiling an SD file

Figure 9 shows an example command line pipe for generating such an SD file. Data are exported from the database using the AEREAExporter program. This command line program has a companion open source web application, AEREA [39], which provides graphical user interfaces for creating search queries and report templates. Search queries and report templates saved in AEREA can be executed from the AEREAExporter program and serve as instructions to retrieve the compounds of interest and data fields specified in the report templates. Thus, AEREAExporter and AEREA enable computational chemists with no knowledge of SQL or the data model to retrieve data from a database for automated processing. The subsequent programs of the example command line pipe perform the following actions:

1. sdfStructureTagger assigns series names to compounds
2. sdfRGroupExtractor generates R-Groups of interest
3. sdfCalcProps calculates physiochemical properties
4. sdfGroovy calculates ligand efficiencies with a custom Groovy script [40]
5. sdfSelectivityCalculator computes off-target selectivity
6. sdfTabMerger adds data from an externally generated tab-delimited file into the SD file.

Piping these command lines provides a certain degree of parallelization because the programs are started simultaneously and process data whenever data are available. The processing time could be further reduced by executing a pipe fragment within sdfMultiplexer that can span multiple threads to parallelize the execution. However, sdfMultiplexer can only be used to parallelize command sequences in which each record is processed independently of the others and for which the input and output order is irrelevant.

The number of project Vortex sessions and their content depends on the need of the given project team. At Genentech, it is the computational chemist’s responsibility to create and maintain the customized Vortex spreadsheets for the project in close interaction with other project team members. The diversity of the command line programs enables the computational chemists to customize the data processing workflows, and thus to provide Vortex spreadsheets that enable analysis from different perspectives. A default Vortex spreadsheet contains compound registration and availability information, aggregated assay results, computed values, such as target selectivity and cell shifts, calculated properties, and possibly includes links to metabolite identification reports, certificates of testing and crystal structures.

Other Vortex spreadsheets combine data exported from DEGAS [41], a “compound idea and synthesis tracking” application with a report of registered compounds. This combined view of “virtual” and existing compounds allows project teams to quickly assess compound ideas based on the experimental results of the registered compounds in the same spreadsheet. Depending on the project team needs, Vortex spreadsheets are generated on a daily or even on an hourly basis using a UNIX computing cluster. The latest exports are easily downloaded from a shared network location via a custom Genentech menu in Vortex.

The Vortex spreadsheets enable the project team to conduct hypothesis-driven drug discovery. Hypotheses evolve in the course of a project’s life and for this reason, the sequence of chemalot command line programs for a project is routinely being adjusted; new assays are added and existing models are changed. Testing and debugging piped command line programs in UNIX can be tedious, especially if the sequence is complex. With the chemalot_knime framework, this can be facilitated by converting the piped programs to a KNIME workflow (Additional file 1: Figure S1) and by using the KNIME Analytics Platform as an editor.

### Quantitative structure–property-relationship (QSPR) model creation

**Fig. 10:**
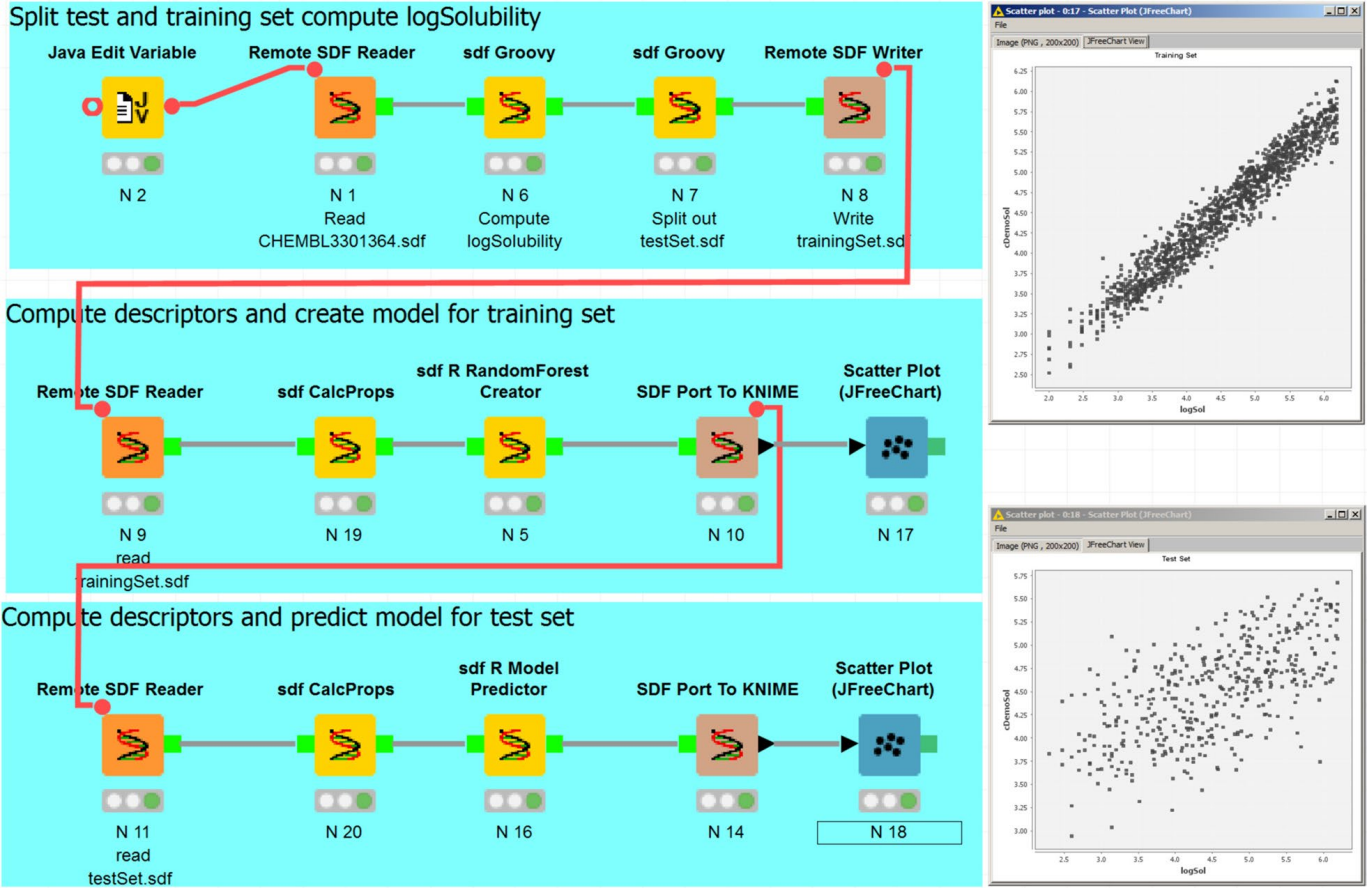
Example QSPR model creation and validation. The KNIME workflow demonstrates the use of the chemalot_knime nodes. It splits the content of the input file into training and test sets, creates a simplified solubility model, and tests the model using the test set. The *scatter plots* on the *right* show predicted (cDemoSol) versus experimental log Solubility (logSol) for the training and test sets

The command line programs and KNIME framework are used at Genentech to create, validate and apply QSPR models for properties important to lead optimization such as metabolic stability [42], permeability [43] and solubility. Here, we present an example KNIME workflow to show how command line programs are used to construct a QSPR model to predict solubility (Fig. 10). The workflow is greatly simplified for clarity and therefore the model should not be used without stringent validation. A dataset with 1763 measured thermodynamic solubility measurements was downloaded from CHEMBL [44, 45]. The workflow first calculates the log10 of the experimental solubility and separates the input file into training and testing sets. In the second step, the descriptors are calculated with sdfCalcProps and the model is built with sdfRRandomForestCreator. The predicted solubility values are converted into a KNIME table and then passed to a KNIME plotting tool to display the experimental versus predicted values. The third sequence uses sdfRModelPredictor to apply the model created previously to the test set. Internally, sdfRRandomForestCreator and sdfRModelPredictor convert the input SD file to tab format and then create models and make predictions using the randomforest package in R [8, 22, 23]. To facilitate the reproduction of this example, the data and the KNIME workflow are available in Additional files 2 and 3, respectively.

At Genentech, predicted drug metabolism and pharmacokinetics (DMPK) properties, e.g. liver microsome clearance, are automatically computed and stored in the database for both registered compounds and compound ideas. To allow users to check the model’s prospective performance, especially for new chemical series, we have implemented web applications using KNIME that apply the models to compounds synthesized after the model creation. The integration of the command line programs in KNIME enables us to leverage the KNIME WebPortal user interface while running the model predictions on our high-performance computing cluster via the chemalot_knime nodes.

### Strain energy analysis

**Fig. 11:**
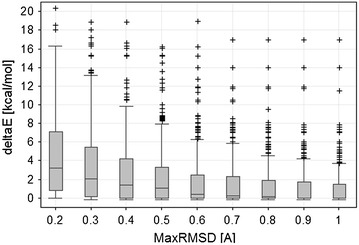
Distribution of strain energies for 416 structures from the PDB. The ∆E is computed relative to the global minimum conformation as described in the sdfMMConfAnalysis section above. Each *box* and *whisker* in the plot represents the same 416 small molecule conformations that were allowed to relax at most the MaxRMSD [Å] amount from the input. The *central horizontal line* shows the median of the values in each *box*. The ends of the *box* show the first quartile and third quartile of the values. The *whiskers* correspond to the highest or lowest point that is not an outlier. Outliers are defined as those points that are outside the median ± 1.5 times the interquartile range

In our final example we demonstrate the use of the sdfMMConfAnalysis program to compute the strain energy of a large number of small molecule protein ligands from the Protein Data Bank (PDB) [46]. Figure 11 shows the distribution of the strain energy for 416 different small molecule conformations retrieved from the PDB database (PDB identifiers are given in the Additional file 4). All small molecule structures with less than 2 Å resolution were exported from the in-house copy of the PDB database stored in PROASIS [47] using sdfExport. The structures were filtered by molecular weight (<500) and number of rotatable bonds (<7) and by removing structures that did not pass our compound screening filters [48]. Finally known metal counter-ions were removed with sdwash [49]. sdfMMConfAnalysis was used to analyze the strain energy for each conformation resulting in multiple conformations with energies for each input as described under “implementation”. For the nine thresholds on the x axis in Fig. 11 (0.2–1), the lowest energy conformation with at most that RMSD distance was retained for each input conformation. For example, if minimized conformers are allowed to deviate by at most 0.4 Å from the input conformation, the median energy relative to the global minimum is 1.42 kcal/mol. 75% of the structures from the PDB have a strain energy of less than 4.25 kcal/mol when they are allowed to relax at most 0.4 Å.

In estimating the strain energy, a certain amount of relaxation from the crystallographic conformation must be allowed for two reasons: The crystallographic conformation is a model optimized to the electron density. The atom positions have associated uncertainties that depend on the resolution of the crystal structure and on the local electron density around the ligand. Secondly, the MMFF94S force field with Sheffield solvation model was used to compute all energies and the force field contains errors. In reviewing the conformations computed for the 416 conformations from the PDB, we decided to use a 0.4 Å RMSD relaxation as a default threshold to estimate the strain energy. 0.4 Å relaxation allows for only a relatively small movement of the atoms as can be seen in Fig. 2. Given this threshold, docking conformations can be filtered or flagged if they exceed a threshold of 4 kcal/mol with a maximum relaxation of 0.4 Å as they are unlikely to have high binding affinity.

The strain energy analysis computed with sdfMMConfAnalysis is regularly used by both computational and medicinal chemists for evaluating strain in new crystal structures and in evaluating new compound designs or docking poses. To facilitate its use, the command line workflow is wrapped as a web service and integrated into the MOE modeling application that is used by all medicinal chemists at Genentech [50].

The three applications presented above and many other command line applications have been in use at Genentech for many years with high impact on small molecule drug discovery projects. The chemalot package was deployed at Denali in mid-2015 and has enabled their computational chemists to support project teams before the deployment of a cloud-based informatics solution. Data files from various contract research organizations (CROs) were easily consolidated for SAR analysis in Vortex. The process of file consolidation, data binning, scaffold matching, molecule clustering, and property calculation was extremely efficient. Command line programs were also used to assess the properties and diversity of the small molecule high-throughput screening (HTS) library from CROs as part of the CRO selection process. Clustering and comparing multiple libraries whose size ranges from 250,000 to 850,000 molecules can be time consuming. With the natural parallel processing of command line pipes plus the divide and conquer feature of sdfMultiplexer.pl, it took a few hours of computing time to process multiple libraries. The results of comparing HTS collections from multiple CROs enabled the project team to make data driven decisions. The chemalot programs are now part of the Denali computational chemistry platform on the Amazon cloud.

## Conclusions

Command line programs can be used for small daily tasks as well as for large distributed computation on a cluster. The chemalot package provides a set of command line programs to many important tasks in chemical informatics. The programs can be easily combined using UNIX pipes to build more complex methods such as the strain energy workflow. The chemalot package extends the autocorrelator and Aestel packages and integrates well with other open source and commercial command line programs.

These packages have been used at Genentech and Denali, a large company and a small startup company, to perform basic and complex cheminformatics tasks that range from merging of structural and numerical data, selecting diverse sets of compounds from a library and, creating machine learning models to performing 3D analyses such as the computation of ligand strain and quantum mechanical torsion scans [51]. At Denali, the command line programs were particularly useful at the start-up stage of the company in 2015 before the deployment of a cloud-based informatics solution. They remain the tools of choice for compute-intensive tasks, such as evaluation of HTS libraries. At Genentech, command line tools are ubiquitous as they are used by computational chemists to generate Vortex spreadsheets for project teams and pharmacokinetic QSPR models for chemical idea evaluations.

The development of complex workflows using piped UNIX command-line programs can be cumbersome. Through the use of automatically generated chemalot_knime nodes, the development of command line pipelines can be significantly simplified. Only five lines of XML configuration are needed to create a chemalot_knime node that encapsulates any UNIX command line program that reads and writes SD files on stdin and stdout. The command line programs can be executed sequentially. The output SD file can be inspected visually in the KNIME user interface and the standard error output can be inspected for each node. Once the workflow has been developed, the chemalot_knime nodes can be executed as single workflow on UNIX, outside of the KNIME environment to optimize performance. Furthermore, conversion nodes enable the integration with other KNIME nodes to leverage the best of these two worlds.

This paper introduces the chemalot and chemalot_knime open source packages and demonstrates their use in two drug discovery organizations. It is our hope that other scientists find these tools useful.

## Additional files

**Additional file 1.** Contains a screenshot of the KNIME workflow corresponding to the example command line pipe shown in Fig. 9 and a list with brief description of each command line program.

**Additional file 2.** Contains the structures and data used to create and validate the solubility QSPR model in Fig. 10.

**Additional file 3.** Contains the KNIME workflow shown in Fig. 10.

**Additional file 4.** Contains the PDB identifiers used to create Fig. 11.

## Abbreviations

CRO: contract research organization
DMPK: drug metabolism and pharmacokinetics
HTS: high-throughput screening
MM: molecular mechanics
MW: molecular weight
PDB: Protein Data Bank
QSPR: quantitative structure–property-relationship
RMSD: root mean square deviation
RO5: rule of five
SD: structure data
SAR: structure–activity-relationship
ssh: secure shell
XML: extensible markup language
