# Supplementary material for: chemalot and chemalot_knime: Command line programs as workflow tools for drug discovery
Source: J Cheminform. 2017 Jun 12;9:38. doi: 10.1186/s13321-017-0228-9 (PMC5468363; doi:10.1186/s13321-017-0228-9)
Supplement: Supplementary file 1 — Additional file 1. Contains a screenshot of the KNIME workflow corresponding to the example command line pipe shown in Fig. 9 and a list with brief description of each command line program. [file 13321_2017_228_MOESM1_ESM.pdf]

Supplement

Following document contains additional details and figures.

Additional figures to "Project Vortex session" example

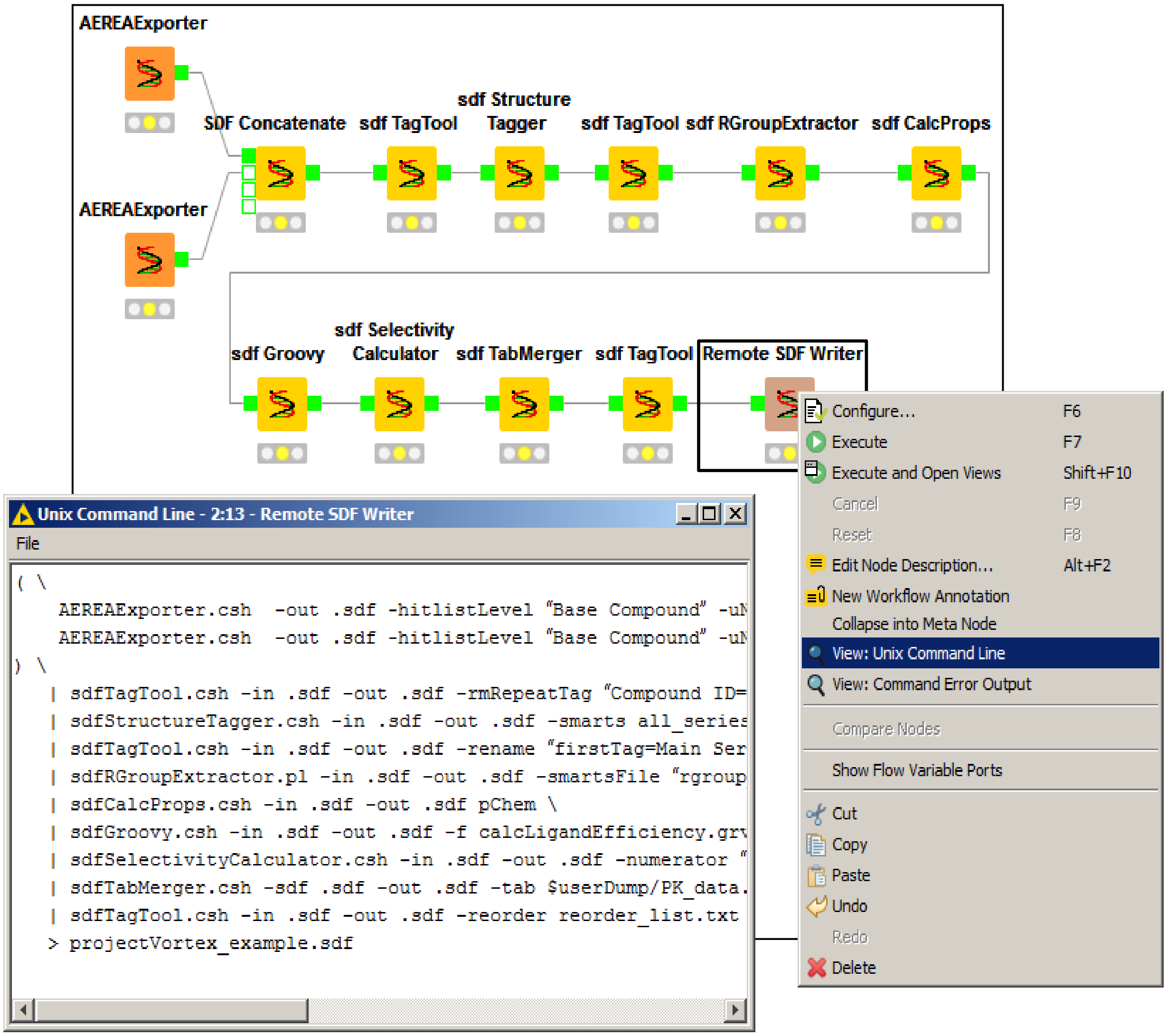

Figure S1. The KNIME workflow contains the command line nodes to generate the example command line pipe shown in Figure 9. The command line text can be retrieved from a node view for further used.

Brief Description of Command Line Programs

(Go to https://github.com/chemalot/chemalot/ for the latest version of the command line program description)

AEREAExporter.csh[1] Export from a database query and report saved using the AEREA application [2,3]

dataLoader.csh[1] Load data into a relational database using the Aestel dataLoader.

g2sdf.pl Create an SDF file from a Gaussian output file.

gOpt.py Optimize using Gaussian enabling faster convergence by computing the second derivatives every 8 steps.

OEProps.csh Compute atom, bond, ring, and other count properties, TPSA, and other 2D properties. (see help text)

qTorsionMultiplexer.pl Perform a Gaussian torsion scan from an input molecule with a specified rotatable bond *Additional requirement: Gaussian*

QTorsionProfileGenerator.csh generate input files for qm torsion file runs

RModelManager.pl Manage models created with sdfR*Creator.pl

sdf2DAlign.csh Transforms the 2D coordinates of input molecules according to the matching substructures specified in the template SDF file.

sdf2g.pl Create Gaussian input files from molecules in an input SDF file.

sdf2Tab.csh[4] convert sdf file to tab separated file

sdfAggregator.csh Given a set of input molecules with SDF tag data, group them by a specified tag value and then perform a grouping function (e.g. find the average "My Assay IC50" (grouping function) for each "Chemical Series" (the group-by SDF tag).

sdfAlign.pl Transforms the 3D coordinates of input molecule conformers to a reference ligand and calculate the RMSD.

sdfALogP.csh Calculates the ALogP and atom type counts [5,6,7].

sdfBinning.csh Groups numerical values into bins

sdfCalcProps.csh Serves as the "properties" warehouse to which any calculator command line programs can be added, thus, enable a single point access to the properties [8].

sdfCatsIndexer.csh Generate CATS fingerprints for input molecules [9].

sdfCFP.csh Generate circular fingerprints for input molecules [10]

sdfCluster.pl Clusters molecules using Atom-Atom-Path similarity and Sphere Exclusion algorithm [11].

sdfConformerSampler.csh Generates conformers combinatorially modifying torsional angles as defined in a torsion file. Torsions of OH and HN2 groups are automatically rotated.

sdfEnumerator.csh Enumerates a combinatorial library given the specified SMIRKS and corresponding reagent input files.

sdfEStateCalculator.csh Compute the occurrence (count) of each E-state atom group in the input molecule and the corresponding sums as well as E-state indices of the individual atoms in a molecule [12].

sdfFilter.csh Remove molecules from the SDF file based on the heavy atom count, number of components, invalid atoms, and max atomic number.

sdfFingerprinter.csh Generate various types of fingerprints including, linear fingerprints and smarts based fragment fingerprints.

sdfFPCluster.pl Use the specified fingerprints to cluster input molecules using the Sphere Exclusion clustering algorithm. A radius of 0.5 is a good value for clustering HTS libraries [13].

sdfFPNNFinder.csh Use the specified fingerprints to identify most similar molecules (nearest neighbors) for each molecule in the input file based on their Tanimoto similarities. It can also be used to compute activity cliffs.

sdfFPSphereExclusion.csh Use the specified fingerprint to compile a diverse sub set using the Sphere Exclusion algorithm [13].

sdfGrep.pl Remove a molecule from the SDF file if the field of interest does not matching the specified requirement

sdfGroovy.csh[4] Apply groovy scriptlet to records in sdf file

sdfLE.grvy Calculate various ligand efficiencies, i.e. LE, LLE [14].

sdfMACCSKeys.csh Generate MACCS keys or counts for input compounds. Based on RDKit [15] and Chemaxon [16] implementations.

sdfMCSSNNFinder.csh User the Atom-Atom-Path similarity to identify the nearest neighbors for the input compounds. It can be used to compute activity cliffs [11].

sdfMCSSSphereExclusion.csh Use MCSS or Atom-Atom-Path similarity to compile a diverse sub set using the Sphere Exclusion algorithm [11].

sdfMDLSSSMatcher.csh Remove molecules from the SDF file that don't match any of substructures in MDL query file

sdfMMConfAnalysis.pl Perform strain energy analysis of input conformers including generation and geometry optimization of a large number of conformations. Also evaluates the energy of the minimized input conformation with several restraint strengths. *Additional requirements: bmin, moebatch, szybki_*

sdfMMMinimize.csh Perform a geometry optimization using a molecular mechanics force field, with wrapped choices of Macromodel (Schrodinger), MOE (CCG) or SZYBKI (OpenEye). *Additional requirements: bmin, moebatch, szybki*

sdfModelCreateValidate.pl Use sdfRModelPredictor.pl to create a Machine Learning Model and validate at the same time using randomly selected training and test sets. Uses sdfR???ModelCreator.pl in the background.

sdfMolSeparator.csh Separate the disconnected molecules in a molfile (e.g. salt and compound) and output them in individual records.

sdfMultiplexer.pl Parallelize the execution of command line scripts by executing multiple instances of a command line string and distributing the input molecules to the various instances. The output is combined back into a single file.

sdfNormalizer.csh Normalize molecules according to Genentech's business rules. Unique tautomers are generated by Quacpac from OpenEye [17]. *Additional requirement: quacpac*

sdfRExecutor.pl Apply custom R scripts to data in SD file and add computed fields to the output file.

sdfRGroupCalcProps.pl Calculate the properties of molecule fragments with attachment points (e.g. [U+1], [U+2]); companion program to sdfRGroupExtractor.pl

sdfRGroupExtractor.pl Fragment input molecules according to the specified transformations in SMIRKS or SMARTS format into R-groups with charged Uranium atoms representing attachment points

sdfRingSystemExtraction.csh Fragments input molecules and outputs the largest (linked) ring system as well as the set of the basic rings as SMILES.

sdfRModelPredictor.pl Compute the prediction according to the specified model created by sdfRRandomForestCreator.pl or sdfRSVMCreator.pl *Additional requirement: R*

sdfRMSDNNFinder.csh Calculates RMSD values between conformers of molecules. This can align conformers by minimizing the RMSD

sdfRMSDSphereExclusion.csh Applies Sphere Exclusion algorithm to find centroids of conformer clusters based on a given RMSD radius

sdfRRandomForestCreator.pl Create models (R sessions) using Random Forest algorithm; companion program to sdfRModelPredictor.pl *Additional requirement: R*

sdfRSVMCreator.pl Create models (R sessions) using Support Vector Machine algorithm; companion program to sdfRModelPredictor.pl *Additional requirement: R*

sdfSdfExport.csh[4] Add data from relational database to sdf file

sdfSdfMerger.csh[4] Merger two sdf files based on common data field

sdfSelectivityCalculator.csh Computes selectivity (ratio) based on the specified numerator and denominator fields considering operator values

sdfSliceByRe.pl Partition sdf files by ranges of rows

sdfSmartsGrep.csh[4] return records that match/do not match a smarts pattern

sdfSorter.csh[4] sort sdf file by one or more data fields

sdfSplicer.csh[4] Get a splice out of an sdf file

sdfStructureTagger.csh Tag molecules with specified names based on the corresponding SMARTS or molfile (queries)

sdfSubRMSD.csh Calculate the RMSD between a supplied fragment (e.g. core) and the matching part of the input molecule. Molecules need to be pre-aligned.

sdfTabMerger.csh[4] Merge sdf file with tab separated file based on common data field

sdfTagTool.csh[4] Perform a large variety of manipulation and filtering based on data in sdf fields

sdfTopologicalIndexer.csh Compute topological indices, i.e. Balaban, Wiener, and Zagreb

sdfTorsionScanner.csh Given one or more molecules (e.g. sdf), generate a set of conformers rotated around a single rotatable bond within the input molecules. Useful as pre-step to sdfMMMinimize.csh to calculate energy torsion scans. (Optional minimization requires a SZYBKI license). *Additional requirement: szybki*

sdfTransformer.csh[4] Apply SMIRKS transformation to chemical structures in sdf file

tab2Sdf.csh[4] Convert tab file to sdf (First column must be SMILES)

tabExport.pl Export data from relational database to tab separated file

tabTagTool.pl Modify column header and filter tab-delimited files

the Software Maintenance Team the Software Maintenance Team

---

1 Implementation supplied by the Aestel jar file.

2 Lee M, Aliagas I, Dotson J, et al DEGAS: Sharing and Tracking Target Compound Ideas with External Collaborators. J Chem Inf Model 2011; 52:278-284.

3 dataLoader and AEREA are open-source applications of Aestel Scientific Information. Contact: aestelSW at gmail dot com

4 Implementation supplied by the autocorrelator jar file.

5 Ghose AK, Crippen GM. J. Atomic Physicochemical Parameters for Three-Dimensional Structure-Directed Quantitative Structure-Activity Relations I. Partition Coefficients as a Measure of Hydrophobicity. Comput. Chem. 1986; 7 (4): 565-577

6Viswanadhan VN, Reddy MR, Bacquet RJ, Erion MD. Assessment of methods used for predicting lipophilicity: Application to nucleosides and nucleoside bases. J. Comput. Chem. 1993; 14 (9): 1019-1026

7Ghose AK, Viswanadhan VN, Wendoloski JJ. Prediction of Hydrophobic (Lipophilic) Properties of Small Organic Molecules Using Fragmental Methods: An Analysis of ALOGP and CLOGP Methods. J. Phys. Chem. A 1998; 102 (21): 3762-3772

8 Feng JA, Aliagas I, Bergeron P, Blaney JM, Bradley EK, Koehler MFT, Lee M, Ortwine DF, Tsui V, Wu J, Gobbi A. An Integrated Suite of Modeling Tools That Empower Scientists in Structure- and Property-Based Drug Design. Journal of Computer-Aided Molecular Design 2015; 29 (6): 511-523.

9 Schneider G, Neidhart W, Giller T, Schmid G. "Scaffold-Hopping" by topological pharmacophore search: a contribution to virtual screening. Angew. Chem. Int. Ed. 1999; 38 (19): 2894-2896.

10 Rogers D, Hahn M. Extended-Connectivity Fingerprints. J. Chem. Inf. Model., 2010;50 (5): 742-754.

11 Gobbi A, Giannetti AM, Chen H, Lee M. Atom-Atom-Path similarity and Sphere Exclusion clustering: tools for prioritizing fragment hits. J. Cheminform., 2015; 7: 11.

12 Hall LH, Kier LB. Electrotopological State Indices for Atom Types: A Novel Combination of Electronic, Topological, and Valence State Information. J. Chem. Inf. Comput. Sci. 1995; 35 (6): 1039-1045.

13 Gobbi A, Lee M. DISE: Directed Sphere Exclusion. J. Chem. Inf. Comput. Sci. 2002; 43 (1): 317-323.

14 Hopkins AL, Groom CR, Alex A. Ligand efficiency: a useful metric for lead selection. 2004; 9 (10): 430-431; Leeson PD, Springthorpe B. The influence of drug-like concepts on decision-making in medicinal chemistry. Nat. Rev. Drug Disc. 2007; 6 (11): 881-890.

15 RDKit [http://www.rdkit.org/]

16 ChemAxon [https://www.chemaxon.com/]

17 Gobbi A, Lee M. Handling of Tautomerism and Stereochemistry in Compound Registration. J. Chem. Inf. Model. 2011; 52 (2): 285-292.
